# Emotions in Times of Pandemic Crisis among Italian Children: A Systematic Review

**DOI:** 10.3390/ijerph20126168

**Published:** 2023-06-18

**Authors:** Aurora Bonvino, Antonella Calvio, Roberta Stallone, Chiara Valeria Marinelli, Tiziana Quarto, Annamaria Petito, Paola Palladino, Lucia Monacis

**Affiliations:** 1 Department of Humanities, University of Foggia, 71121 Foggia, Italy; aurora.bonvino@unifg.it (A.B.); antonella.calvio@unifg.it (A.C.); chiaravaleria.marinelli@unifg.it (C.V.M.); tiziana.quarto@unifg.it (T.Q.); paola.palladino@unifg.it (P.P.); 2 Department of Clinical and Experimental Medicine, University of Foggia, 71121 Foggia, Italy; roberta.stallone@unifg.it (R.S.); annamaria.petito@unifg.it (A.P.)

**Keywords:** emotion, children, Italy, lockdown, social isolation, risk factors, systematic review

## Abstract

Several studies underlined the negative effects of forced social isolation on emotional processes in younger population. The current study aimed to review existing evidence of the pandemic’s impact on the emotional regulation of Italian children aged 0–12 years in order to identify personal and contextual factors that may adversely impact their developmental process. Different electronic databases (Web of Science, APA PsycInfo, APA PsycArticles, MEDLINE, Psychology and Behavioral Sciences Collection, and Scopus) were used to identify peer-reviewed studies published in English and Italian. Thirteen studies were included in the review, covering a total of 18.843 children. All studies reported negative effects of the lockdown on a child’s emotional processes. The most affected were children aged 3–5 years, those living in Northern Italy, and those with low socioeconomic status (SES) families. Alterations in emotional processes were associated with sleep disturbances, quality of family relationships, personality structures, the coping strategies used, and time spent with technological devices. Finally, two- (time × parenting) and three-way (time × parenting × environmental sensitivity) interactions resulted significantly in predicting a child’s emotional regulation, respectively, in terms of externalizing and internalizing behaviors. This review remarks that children’s emotional processes were negatively impacted during social lockdown, especially where acute social isolation interacted with a set of dispositional and situational risk factors.

## 1. Introduction

Among the negative effects of COVID-19 on mental health, current studies have been highlighting alarming emotional distress across the lifespan in the post-lockdown period among children with pre-existing psychopathological problems [1] or without any previous signs of psychopathological risk [2]. During childhood, the interaction between innate traits and life events increasingly concurs to organize the outcomes of the growing process [3]. Although stress response is not specific or predetermined (that is not all stressed people develop stress-related symptoms) [4], stressful events not only impact healthy development [5,6] but also impair brain function. Prior research reported the effects of exposure to stressful events on the integrity of the cortico–limbic circuit connections that involve emotion regulation-related abilities, impulse control, and social intelligence [7], and on the long-term alterations in the brain [8].

Evidence of brain plasticity related to volume change patterns has been provided in healthy adults, who were not somatically affected by COVID-19 infection following the real-life external event of the global pandemic. The high rate of psychological distress (stress and anxiety) attributed to social effects of the COVID-19-related lockdown, such as social isolation and perceived uncertainty, seemed to be associated with the transient volumetric enlargement of some brain regions (amygdala, putamen, and ventral anterior temporal cortices) [9]. In addition, a recent fMRI study [10] focused on neural correlates of psychological general distress and showed that some brain features prior to the COVID-19 epidemic could predict the later emergence of distress, mainly including within and between the default mode network (DMN) connection patterns, with the left hippocampus emerging as the most critical hub region. The identified susceptibility markers considered, as fingerprints of individual differences toward pandemic-related distress symptoms/alterations among young adults may serve as targets for early psychological and/or brain intervention, such as mindfulness-based training programs and neurofeedback procedures, to embark on long-term coping strategies in terms of limited mental healthcare resources. However, little is known about the effects of the viral infection itself and the secondary effects of COVID-19-related stressors on ongoing brain dysfunction in children, suggesting that much research is needed in this area.

When shifting from the organic brain alterations to psychological alterations, an exponential increase in both emotional and behavioral dysregulation has been observed across younger populations [11]. Indeed, the introduction of safety measures to contain the spread of the virus has led to social/physical distancing and sudden lifestyle changes (e.g., deprivation of social contact with peers and isolation, school closures, and distance learning) that have negatively affected quality of life, mental health, and learning [12]. An increase in adverse mental and behavior outcomes, such as impulsivity, aggression, anxiety, mood deflection, sleep-related problems, eating habits alterations, widespread academic decline, and smartphone overuse, have also been recorded [11,13,14]. Similarly, in the Italian context, Uccella and colleagues [15] have shown that children and adolescents between 6 and 18 years suffered from experiencing considerable discomforts and adverse behavioral outcomes, such as emotional instability, irritability, mood changes, and alterations of sleep-wake rhythm. Put differently, externally mandated social isolation has been mentally and physically aversive for children and adolescents, who have experienced an unprecedented social craving. The observed results could be aligned with current neuroimaging studies on the underlying neural mechanisms of unmet social needs, such as loneliness. Individuals forced to be isolated crave social interaction in the same way in which a hungry person craves food. Therefore, empirical evidence has been provided for the analogy that acute isolation causes social craving, similar to the way fasting causes hunger [16]. 

During the epidemic period, other forms of communication, such as virtual communication, have been encouraged in the hope that they could be a valid alternative for promoting social bonding and affiliative behaviors. Prior biological and neuroscience investigations provided evidence for the positive effects of social relationships on distressing and physical responses. Indeed, previous investigations based on neuroendocrine methods documented the positive effects of social integration and social support on reward-relevant and anxiety-reducing structures and transmitter systems, which can, in turn, reduce biological stress reactivity [17,18]. Furthermore, neuroimaging studies indicated that social support reduces activity in brain regions implicated in emotion regulation (i.e., anterior cingulate cortex, dorsolateral and ventrolateral prefrontal cortex), when social embodied support is provided [19,20]. Despite the use of technology in preserving social connection and support, data on the effects of the lockdown reported generally adverse emotional health among adolescents and children, probably because of the touch deprivation in social relations [21]. Indeed, being that the touch experience is a powerful mechanism of non-verbal communication for the formation and maintenance of social bonds, the ability to receive this type of social support has probably been affected by the physical distancing regulations and long-term isolation at home, thus determining social touch craving and, therefore, struggles in emotion regulation, sleep problems, and school performance.

Defined as the process of understanding, monitoring, and expressing internal states in adaptive and functional ways, emotion regulation is a key element in the well-being of individuals. However, limited studies have examined emotion regulation in the context of COVID-19, and they are mainly focused on the effects of the tendency to express emotions in an impulsive and disorganized way in adult populations [22,23]. Conversely, little attention has been paid to emotional dysregulation during the lockdown in children. 

The Italian scenario has been particularly dramatic in terms of mortality. Indeed, according to the European Center for Disease Prevention and Control (2020), Italy has been the third country in the world for the number of positive cases and the second in the world for the number of deaths. To deal with the sanitary emergency, the Italian government imposed staying-at-home and social distancing by establishing two national lockdowns (March–May 2020 and October–November 2020) and a three-tiered system of restrictions based on the combination of different quantitative indicators, such as the level of transmission, the burden on older age groups and healthcare, and resilience of monitoring systems. Such restrictions characterized an atypical and prolonged stressful life experience relevant especially for children and adolescents, thus leading to repercussions on public health problems in terms of children and adolescents’ quality of life and school performance. 

Indeed, when looking at age-related differences in emotions, cognitive attitudes, and behavioral responses to the COVID-19 crisis, Ceccato et al. [24] found empirical evidence for the Socio-emotional Selectivity Theory (SST) [25], according to which older adults, being more present-focused and more oriented toward positive emotions and meaningfulness in life, were moderately more optimistic than young and middle-aged adults, and showed efficient emotional regulation strategies that allowed them to focus on positive emotions and to reduce negative affect. Similarly, Maggi et al. [26] reported that younger adults showed higher levels of anxiety and anger symptoms rather than middle-aged individuals. 

In the context of children’s development, the cumulative risk hypothesis has been applied to examine different development outcomes. According to this hypothesis, the greater the number of risk factors, the greater the prevalence of clinical problems; that is, the increasing number of concurrent risk factors yields a cascading, deleterious effect on later developmental outcomes on the basis of a quadratic (i.e., multiplicative) or linear (i.e., additive) effect [27]. Furthermore, the interactions of socio-demographic, psychological, parental, and contextual variables can act as risk factors for internalizing (i.e., anxiety) and externalizing (i.e., aggressive behavior) symptoms of emotion dysregulation in childhood [28], and, as already demonstrated, children who experienced internalizing and/or externalizing problems are more likely to experience a wide range of psychiatric disorders [29] and long-lasting psychosocial problems in adulthood [30]. Following the developmental psychopathology perspective, the cumulative nature of risk factors on a child’s emotion regulation adjustment has been examined during the Italian lockdown by Spinelli and colleagues [31], who reported that children were more vulnerable to maladaptation when they experience multiple (co-occurring) risks as opposed to a single COVID-19-related risk factor. 

However, according to the authors’ knowledge, no systematic studies focused on the Italian context have analyzed the deleterious effects of co-occurring risk factors on behavior outcomes incurred in early and middle childhood in times of the pandemic. Thus, understanding interactions between all possible psychosocial variables implicated during the COVID-19 pandemic is imperative for policymakers to guide policy surrounding public health, as well as for clinicians and educators to guide treatment and intervention and to develop effective well-being strategies. In light of these premises, the following review aims to bring together the evidence of the lockdown effects on Italian children’s emotional dysregulation that could negatively impact their developmental trajectories. 

The objectives of the current review were to (i) conduct a systematic literature search on studies investigating the effects of the lockdown on emotional processes among Italian children in all areas of their daily functioning; (ii) evaluate the methodological quality of these studies; (iii) synthesize the results on risk factors affecting emotional regulation. 

In light of these objectives, the current review intended to answer the following research questions:

RQ1: What are the effects of the lockdown and post-lockdown period on Italian children in terms of emotional problems?

RQ2: What are the risk and protective factors associated with increased vulnerability in emotional response?

The answers will provide insight into the potential harm of emotional dysregulation on children’s development and inform policymakers on how to promote the best practices to better protect vulnerable children from short- and long-term emotional distress.

## 2. Materials and Methods

The current review, including the research questions, search strategy, inclusion and exclusion criteria, and risk of bias assessments was conducted according to the guidelines for the Preferred Reporting Items for Systematic Reviews and Meta-Analyses (PRISMA) checklist that included 27 items to be observed when reporting on literature and systematic reviews [32,33]. The study was preregistered on the Open Science Framework (https://doi.org/10.17605/OSF.IO/N2BQZ, accessed on 20 January 2023).

### 2.1. Inclusion Criteria

When deciding whether a study should be included in the current review, a set of criteria to be met was applied. Each study had to be carried out on the Italian population; the sample had to include children population aged 0 to 12 years; participants had to not be diagnosed with a neurodevelopmental disorder (or other disorders such as ADHD, autism, etc.); only studies written in English and Italian and published in peer-reviewed journals were taken into consideration; each investigation had to operationalize emotional processes on the basis of objective or subjective (e.g., self-reports or perceived) measures and had to examine (short- and long-term) lockdown effects on emotional child process; and studies obviously required a publication date between 2020 and 2023. Studies were excluded from the review if they were editorials, reviews, dissertations, theoretical or qualitative studies, single-case studies, if their full text was not available, and if they did not meet the mentioned inclusion criteria. 

### 2.2. Search Strategy and Screening Process

Web of Science, APA PsycInfo, APA PsycArticles, MEDLINE, Psychology and Behavioral Sciences Collection, and Scopus were used as platforms to run a multi-database search (from the beginning of the Italian quarantine 2020 to date—March 2023). The research terms were (“Quarantine” OR “self-isolat* OR “lockdown” OR “lock-down”) AND (“Ital*” OR “Italian”) AND (“child*” OR “children” OR “infant*”) AND (“emotion*” OR “emotional problems” OR “emotional regulation” OR “emotional dysregulation” OR “emotional distress” OR “emotional well-being”). Different search strategies were scrutinized, and the final strategy that obtained the highest number of relevant studies was used for each individual database. The search results were imported using Microsoft Excel. The initial literature search provided 911 potentially relevant records. Then, duplicates were screened and removed, thus identifying the records that were retained and screened by applying the inclusion criteria. All identified records were blinded (i.e., the reviewers were blind to each other’s assessments) and screened for potential relevance based on the title and abstract by three independent researchers (AB, AC, RS). Disagreements were discussed and resolved. After these initial screening steps, full texts were independently reviewed by the first three authors, taking into account the specific reference to the eligibility criteria. To reach a joint decision, the emerging discrepancies were discussed within the team via an online discussion. In addition, an inter-rater reliability analysis was then performed by using the Fleiss Kappa (κ) test, which is a measure of the agreement between more than two raters, whose values indicated the level of agreement [34]. The results of the Fleiss Kappa analysis provided a coefficient, κ = 0.670, z = 0.217, *p* < 0.001, which indicated substantial agreement among raters when reviewing full texts for inclusion in the final review [29].

### 2.3. Data Extraction, Strategies for Data Synthesis, and Quality Appraisal

The first author extracted the data from the eligible studies using an Excel spreadsheet, and a second reviewer (RS) checked these data. The following coding process was followed: (1) lead author/year, (2) key ideas of the research, such as key characteristics of sample size, mean age, sex, geographic area, measures of emotional variables, reports (parent or child version), period of data collection, and (3) key findings, such as correlation and regression coefficients and mean difference estimates. 

The results of the systematic review were summarized in tables and discursively synthesized, highlighting the effects of the lockdown on children and identifying the variables most associated with dysfunctional developmental trajectories. Therefore, the results were stratified by poor emotions, mental health, and behavioral outcomes in children and parents.

The Newcastle–Ottawa Scale (NOS) for cohort studies [35] and its adapted version for cross-sectional studies [36] were used to assess the quality of the evidence and risk of bias in the longitudinal and cross-sectional studies included in the present review, respectively. The first tool comprises eight categories mapped onto three domains: selection, comparability, and outcome. The first domain includes four categories: representativeness of the exposed cohort, selection of the non-exposed cohort, the ascertainment of the exposure, and the demonstration that the outcome of interest was not present at the start of the study, with ratings ranging from 0 to 4 stars. The second domain comprises the category of the comparability of cohorts based on design or analysis, with ratings ranging from 0 to 2 stars, and the last domain refers to the assessment of the outcome, the follow-up length, and the adequacy of the follow-up, with ratings ranging from 0 to 3 stars. A total score is calculated ranging from 0 to 9 stars for each study. The instrument classifies the study into 4 possible levels: very good (9 points), good (7–8 points), satisfactory (5–6 points), and unsatisfactory (0–4 points).

The second tool, the NOS-adapted version for cross-sectional studies, comprises seven categories mapped onto the same three domains. The first domain includes four categories: representativeness of the sample, the sample size, the number of non-respondents, and the ascertainment of the exposure, with ratings ranging from 0 to 5 stars. The second comprises the category of the control for confounding factors with ratings ranging from 0 to 2 stars, and the last domain refers to the outcome and the appropriate usage of statistical tests with ratings ranging from 0 to 3 stars. A total score is calculated ranging from 0 to 10 stars for each study. The instrument classifies the study into 4 possible levels: very good (9–10 points), good (7–8 points), satisfactory (5–6 points), and unsatisfactory (0–4 points).

## 3. Results

### 3.1. Literature Search and Selection Process

**Figure 1:**
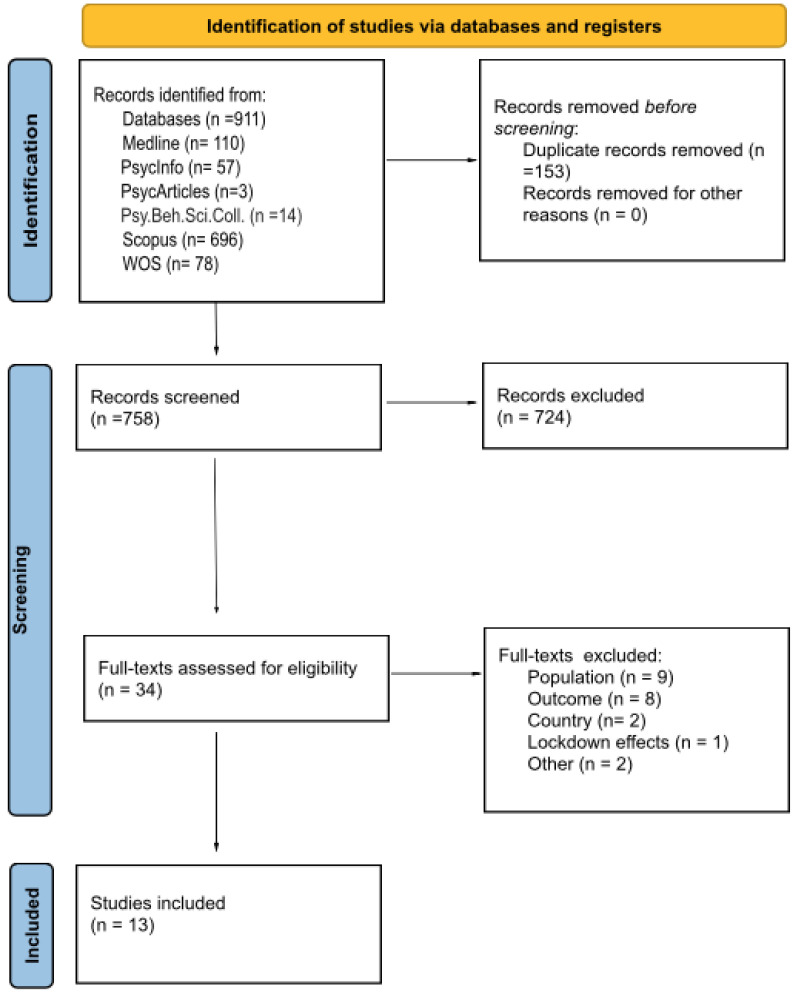
PRISMA flowchart.

The initial searches performed for the literature review provided 911 records. Duplicates were removed (N = 153), and 758 entries were screened. After screening titles and abstracts based on the relevance of the current topic, 724 articles were removed, thus leaving a total of 34 articles for the assessment of the eligibility criteria. This led to the exclusion of 22 reports in line with the inclusion criteria. After reviewing the full texts, nine articles were excluded since the samples included a population over 12 years, from adolescents to young adults [2,37,38,39,40,41,42,43,44]; eight studies focused on wrong outcomes [45,46,47,48,49,50,51,52]; two studies enrolled children living outside Italy [53,54]; one study was not focused on the variables within the context of the lockdown effects [55]; and two studies were excluded for other reasons, i.e., the first recruited participants had a pre-existing neuropsychiatric diagnosis [56] and the second one reported only qualitative data [57]. As a result, a total of 12 articles, including 13 studies, were eligible for inclusion in the qualitative assessment [58,59,60,61,62,63,64,65,66,67,68,69]. Figure 1 depicts the PRISMA flowchart of the literature search process. 

### 3.2. Quality Appraisal

The quality appraisal of the 13 studies is reported in Table S1 (Supplementary Material). The overall mean value of the ten cross-sectional studies [58,59,60,61,63,64,65,66,68,69] was good with 7.1 stars. Three studies [58,59,64] were at the satisfactory level since they obtained values equal to 6, and seven studies [60,61,63,65,66,68,69] were in the good level since they obtained values ranging from 7 to 8 as total scores). The overall mean value of the quality check for the three longitudinal studies [62,67] was satisfactory with 5 stars. All four studies were at the satisfactory level since they obtained values equal to 5. 

Consequently, the quality of the current review can be considered to be a good level considering cross-sectional studies, with an average of the selection domain equal to 4.2 out of 5 stars, an average of the comparability domain equal to 0.9 out of 2 stars, and an average outcome domain equal to 2 out of 3 stars. Finally, the quality of the current review can be considered to be a good level considering longitudinal studies, with an average of the selection domain equal to 2 out of 4 stars, an average of the comparability domain equal to 1 out of 2 stars, and an average outcome domain equal to 2 out of 3 stars.

### 3.3. Sample Characteristics and Demographic Information

Table 1 shows the sample and demographic information. The sample sizes ranged from N = 72 to N = 9688. In terms of gender proportions, six studies [60,61,64,65,66,69] had more male participants than females (i.e., >52% males), and seven studies [58,59,62,63,67,68] had more females than males (i.e., >52% females). Across studies, the distribution of the sample according to gender was 52% males and 48% females. Age was reported as an ordinal variable across ten of the twelve studies with mean values ranging from 3.82 to 9.08 years and standard deviations ranging from 1.38 to 0.56. The study carried out by Oliva et al. [65] considered infants separately (<1 years), preschool children (1–6 years), and primary and middle school children (6–12 years). Picca and colleagues [66] categorized age into two groups with a range of 5 (young 1–5 years vs. old children 6–10 years). 

### 3.4. Study Design and Sampling Methods

Ten studies included in the review were cross-sectional in design [58,59,60,61,63,64,65,66,68,69] and three studies were longitudinal [62,67]. Nine studies recruited parents through online surveys distributed in schools and used social media platforms and a snowball sampling strategy. They were asked to self-report their children’s psychological responses and daily habits [58,59,60,61,62,63,65,66,68]. Two studies recruited mothers from hospitals: one study at antepartum classes or immediately after the postpartum period [67] and the other through a flyer distributed in hospitals before the pandemic period [62]. One study collected children’s self-report responses in classrooms rather than online [64]. Finally, one investigation interviewed both school children (with video registration) and their parents (with an online self-reported questionnaire) through a collaboration with the school [69].

Regarding the geographic areas, 67.60% of the children came from Northern Italy, 16.86% from Central Italy, and the remaining 15,54% from Southern Italy and the islands. In particular, four studies [59,60,65,68] have considered children from all over Italy: one study [58] from Northern and Central Italy, three studies [58,64,66] from Northern Italy (Piemonte, Friuli Venezia Giulia, and Lombardy, respectively), and five studies did not report the geographic areas of the participants’ residence [62,63,67,69]. 

As for the data collection period, six investigations [59,60,61,63,65,69] reported the effects about one month after the restrictive measures, three studies [58,66,68] focused on the outcomes observed during the COVID-free period (summer of 2020), three studies investigated the effects of the acute isolation experience in the first wave [62] (study 1 and study 2) and between the first and the second wave [67], and the last one [64] dealt with the effects that occurred in the second wave (October–November 2020).

### 3.5. Measurement and Psychometric Assessment of Emotions

The reported emotional outcomes are heterogeneous among the selected studies. Four studies used the parent version of an ad hoc online questionnaire to measure emotional abilities in children. Indeed, by investigating the risk of maladjustment of children and families during the lockdown period, Arace and colleagues [58] developed a 33-item battery focusing on the child’s behavioral and emotional problems perceived by parents. Similarly, in dealing with childhood insomnia, Bacaro et al. [59] asked parents to rate, using a rating scale from 1 to 100, how happy, sad, anxious, angry, or quiet their children were during the lockdown. Mariani et al. ’s study [63], which examined the role played by parental resources and resilience processes in the association between child resilience and stress-related behaviors during the lockdown, formulated an ad hoc list of eight stress-related behaviors (difficulty standing still, concentration difficulties, nervousness and irritability, tendency to cry for no reason, difficulty falling asleep, restless sleep with awakenings, food refusal, and excessive food seeking) and asked parents to indicate the presence of each behavior before the COVID-19 outbreak and during the confinement period.

The study carried out by Picca et al. [66] analyzed parental well-being and children’s psychological health by asking parents to express their perception of their children’s possible changes in daily life, behaviors, relationships, technological devices, and distance learning experience during the COVID-19 pandemic in terms of the different frequency of their children’s irritability, mood and sleep disturbances, and attention deficits on tasks.

The remaining studies used validated and/or adapted versions of parental or child reports to evaluate emotional factors. Indeed, Cellini et al. [66] examined the interplay between children’s (6–10 years old) sleep and changes in daily routine to predict their emotional symptoms and difficulties, taking into account changes in mothers’ sleep and the resulting emotional symptoms. To measure emotional dysregulation, three validated questionnaires were used: (i) the Strengths and Difficulties Questionnaire (SDQ-P), a parent report screening tool, to evaluate children’s difficulties with three subscales, emotional symptoms (five items), hyperactivity inattention (five items), and the conduct problem (five items); (ii) the Strengths and Difficulties Questionnaire-18+ (SDQ-18+), a screening self-report questionnaire for parents’ difficulties, with the same subscales selected in the previous instrument; (iii) and the Difficulties in Emotion Regulation (DERS), a self-report tool used to capture mothers difficulties in emotion regulation. The first and second tools were completed twice to compare scores obtained from home confinement with the pre-confinement period. The last tool, which is a trait-based measure of emotion dysregulation, was referred to as home confinement and was completed only one time.

The investigation carried out by Liang et al. [61] mainly focused on the psychosocial impact of COVID-19 in children using an online survey sent to parents that included two tools: (i) an adapted version of the Impact Scale of COVID-19 [70] composed of 24 items on a five-point scale, ranging from 1 (much less compared to before quarantine) to 5 (much more compared to before quarantine), to measure children’s psychological responses to quarantine according to 24 symptoms grouped into four categories: anxiety symptoms (10 items), mood symptoms (6 items), behavioral changes (6 items), and cognitive changes (2 items); and (ii) a list of 11 items translated into Italian that comprised the three coping strategies for childran, i.e., task-oriented, emotion-oriented, and avoidance-oriented, according to Parker and Endler’s (1992) model [71].

Lionetti et al.’s studies [62], which aimed at investigating the interplay between parenting and environmental sensitivity in predicting children’s externalizing and internalizing behaviors during COVID-19, used different tools for measuring the externalizing and internalizing behaviors on the basis of child’s age. For preschoolers aged between 1½ and 5 years old, the Child Behavior Checklist (CBCL) was used. For schoolers, a short version of the Pediatric Symptoms Checklist (PSC) with three items for externalizing behaviors (“fights with others”; “does not listen to rules”; “teases others”) and three items for internalizing behaviors (“feels sad, unhappy”; “is down on him/herself”; “worries a lot”) that were translated into Italian following standard translation–back translation procedures were used. Both instruments were completed by parent figures.

The study conducted by Matiz and colleagues [64] aimed (i) to explore the impact of the pandemic on children’s effects and personality development and (ii) detect any differences by comparing the observed scores that emerged during the pandemic period with the normative scores related to the same variables collected before the pandemic. For this purpose, children were recruited from primary schools and were asked to evaluate their positive and negative effects experienced in the previous weeks during the second wave, their general concordance/discordance with each statement of the personality dimensions, and finally, their agreement/disagreement on feelings of fear toward COVID-19. All statements were reported in a paper–pencil questionnaire composed of validated self-reported questionnaires, such as the children’s version of the Positive and Negative Affect Scale (PANAS-C) for positive and negative affect, the junior version of the Temperament and Character Inventory (jTCI) for personality dimensions, and the Fear of COVID-19 scale (FCV-19S).

Oliva and colleagues [65] analyzed risk and protective factors for child’s adverse psychological health by using different age-standardized screening instruments for the emotional and behavioral assessment. For toddles, the Baby Pediatric Symptom Checklist (BPSC) with three sub-scores: inflexibility, irritability, and routine; for preschoolers (1–6 years), the Preschool Pediatric Symptom Checklist (PPSC); and for schoolers (≥6 years), the Pediatric Symptom Checklist (PSC). In addition, the Center for Epidemiological Studies Depression Scale for Children (CES-DC) and the Screen for Child Anxiety Related Disorders (SCARED) were also used to assess depressive and anxiety symptoms in children aged 6 years and above. For infants and preschoolers, the instruments were completed by parents.

Provenzi et al.’s [67] longitudinal investigation examined the consequences of pandemic-related prenatal stress on infants’ regulatory capacity, which was assessed by the short-form version of the infant behavior questionnaire—revised, IBQ-R. The instrument comprises the following dimensions of infants’ temperament: cuddliness, duration of orienting, low-intensity pleasure, and soothability. A total score was calculated as an index of the infant’s regulatory capacity.

Scaini and colleagues [68] identified children’s different temperament profiles as potential risk factors for the development of psychopathological symptoms and low levels of resilience. To measure the child’s mental health problems and psychological adjustment, the parent version of the Strengths and Difficulties Questionnaire (SDQ-P) was used. It consists of 25 items on a 3-point Likert scale and it is divided into five scales: emotional symptoms, conduct problems, hyperactivity inattention, peer problems underlying difficulties, and prosocial behavior underlying strengths. Three scores were reported: the internalizing symptoms score resulting from adding emotional symptoms to peer problems; the externalizing symptoms score obtained from adding conduct problems and hyperactivity inattention, and a total score resulting from the four scales of the difficulties.

Scrimin et al. [69] conducted an exploratory study on children’s emotional and physical health in response to the COVID-19 pandemic by using the Emotional and Physical Comfort subscales belonging to the Child Health and Illness Profile—Child Edition (CHIP-CE). The self-reported questionnaire evaluates the child’s health status and well-being. The children were invited to report the frequency of emotional and physical symptoms experienced in the past month (“how often did you feel very sad” or “ how often did you have a bad stomachache” on a 5-point scale (0 = never, 4 = always).

### 3.6. Emotional Alterations in Children

The following sections were clustered according to the levels of increased negative emotions experienced during the data collection (before, during, and post-lockdown period), the associations of emotional factors with behavioral outcomes (sleep disturbance), parent–child relationships, child dispositional factors and coping strategies, and technological abuse.

#### 3.6.1. Emotional Alterations and Mental Health

Five studies ran mean difference analyses on children’s emotional processes and reported a significant negative effect of quarantine. Of these, the first study [58] reported remarkable differences in the three time assessments (before, during, and post-lockdown period), generalized anxiety (GA) (F_1,942_ = 66.68, *p* < 0.001), and anxiety when faced with novelty (AN) (F_1,942_ = 29.34, *p* < 0.001). As expected, children showed higher levels during the lockdown period (for GA, M_males_ = 1.57, SD = 0.79 and M_females_ = 1.55, SD = 0.77; for AN, M_males_ = 1.93, SD = 0.85 and M_females_ = 1.75, SD = 0.82). In addition, lower levels were observed in the post-lockdown phase (for GA, M_males_ = 1.43, SD = 0.71 and M_females_ = 1.39, SD = 0.66; for AN M_males_ = 1.79, SD = 0.78 and M_females_ = 1.61, SD = 0.73), although they were higher than those reported in the baseline measurement (before the lockdown period, for GA, M_males_ = 1.33, SD = 0.59 and M_females_ = 1.31, SD = 0.56; for AN, M_males_ = 1.73, SD = 0.71 and M_females_ = 1.64, SD = 0.73). Moreover, during the lockdown period, 38.80% and 32.80% of children needed more time being held and having an adult sleep with them, respectively.

The investigation carried out by Bacaro and colleagues [59] portrayed the intensity of children’s moods experienced during the lockdown period on the basis of parents’ rates and showed that compared to other age groups (aged 3–5 and 6–12 years), children aged 0–2 years were happier, sadder, and more anxious (M = 74.5, SD = 19.9; M = 33.9, SD = 31.8; M = 28.9, SD = 31.18). In addition, in all age groups, the score in the mood “Angry” and “Calm” was similarly quite high.

The third study [60] compared the mean values of retrospective data with those collected during the first months of the lockdown on the three subscales of psychological difficulties, i.e., emotional symptoms (EMO), hyperactivity inattention deficits (HYPER), conduct problems (COND), and the total score of psychological difficulties (PDS). The results indicated a trend of worsening in EMO (F_1,291_ = 3.84, *p* = 0.051), a significant increase in COND (F_1,291_ = 28.17, *p* < 0.001), HYPER (F_1,291_ = 16.31, *p* <0.001), and the total score of the PDS (F_1,291_ = 24.74, *p* < 0.001). In addition, findings showed that mothers’ difficulties in emotion regulation covaried positively with the increase in EMO (F_1,289_ = 14.29, *p* < 0.001; coefficient = 0.02, SE = 0.005, t = 3.78), COND (F_1,289_ = 14.68, *p* < 0.001; coefficient = 0.02, SE = 0.005, t = 3.83), HYPER (F_1,289_ = 8.10, *p* < 0.010; coefficient = 0.01, SE = 0.004, t = 2.85), and in general with PDS (F_1,289_ = 22.10, *p* < 0.001; coefficient = 0.05, SE = 0.01, t = 4.70).

The fourth study [61], by comparing psychological and emotional outcomes in children living in Northern and Central Italy, showed that children from northern areas were more affected than those from central ones (93.1% vs. 87.1%; χ^2^ = 9.245, *p* < 0.010). In addition, higher levels of anxiety (U = 93963, *p* < 0.001), mood alteration (U = 101269, *p* < 0.010), and cognitive symptoms (U = 105596.5, *p* < 0.050) were also reported. Indeed, significant differences emerged between the two groups: compared to children from Central Italy, their counterparts seemed to be more worried (χ^2^ = 23.120, *p* < 0.001), more preoccupied with death (χ^2^ = 10.684, *p* < 0.001), more easily alarmed (χ^2^ = 9.074, *p* < 0.010), more afraid of COVID-19 infection (χ^2^ = 17.347, *p* < 0.001), more concerned when someone left the house (χ^2^ = 34.865 *p* < 0.001), and more bored (χ^2^ = 24.817, *p* < 0.001), and they seemed to be sadder (χ^2^ = 8.621, *p* < 0.010). In terms of cognitive changes, children in the northern areas showed more difficulties concentrating (χ^2^ = 4.541, *p* < 0.050). Finally, although no significant difference emerged in the total score of behavioral changes, the findings indicated that children from central areas were more likely to be quiet (χ^2^ = 5.275, *p* < 0.050) and less angry (χ^2^ = 4.159, *p* < 0.050) in comparison with those from Northern Italy.

Finally, the last study [65], which focused on changes in emotional and behavioral status before and during the lockdown, showed an increase in the symptoms during home confinement ranging from 35% for infants < 1 year to 73.3% for preschoolers, and from 40.5% for primary schoolers to 60% for middle schoolers.

#### 3.6.2. Effects of Gender and Age on Emotional Problems

One study [58] observed a main effect of gender during and after the lockdown on emotional externalizing problems. Males were more hyperactive (F_(1,942)_ = 5.29; *p* < 0.050), more nervous and aggressive (F_(1,942)_ = 5.29; *p* < 0.005), and showed more sleep problems and anxiety than females (F_(1,942)_ = 6.25; *p* < 0.050). The authors also observed an interaction between gender and time (during and after lockdown). Males were significantly more nervous (F_(1,942)_ =13.99; *p* < 0.001) and hyperactive (F_(1,942)_ = 25.95; *p* < 0.001) and showed more difficulties in separation from parental figures (F_(1,942)_ = 4.68; *p* < 0.050) than females after the lockdown.

Two studies [58,65] have identified preschoolers (age range of 3–6 years old) as the subjects most affected in terms of emotional diseases. During the lockdown, an increase of 35% has been observed for children < 1 year, 73.3% for preschool children, and 40.5% for primary school children in terms of emotional internalizing problems (anxiety and depression) [65]. Children between 3 and 6 years showed more problems in terms of regressive behaviors (F_(2,804)_ = 8.573; *p* < 0.001) and emotion regulation (F_(2,897)_ = 4.154; *p* < 0.01) and reported higher levels of fear or hesitation toward habitual activities (F_(2,916)_ = 3.300, *p* < 0.050) compared to 1–3 year subjects [58].

#### 3.6.3. Association between Emotional Dysregulation and Sleep Disturbances

Two studies have investigated the relationships between alterations in emotional processes and sleep disturbances during the pandemic period. The former [59] reported a trend of an increase in insomnia in children living in the North of Italy compared to those living in other parts of Italy (OR = 1.23, 95% CI [0.98–1.55], *p* = 0.071) and identified predictors of insomnia. In addition, data demonstrated that a younger age was more associated with childhood insomnia-related symptoms (OR = 0.88; *p* < 0.001) and that some negative emotions were significantly associated with the prevalence of insomnia symptoms, even if in the opposite direction. While the mood “Sad” was associated with less insomnia (OR = 0.93, CI [0.88–0.98], *p* < 0.050)], the moods “Anxious” and “Angry” were found to be significantly linked to a higher prevalence of insomnia (OR = 1.06, 95% CI [1.00–1.11], *p* < 0.050 and OR = 1.11, 95% CI [1.07–1.17], *p* < 0.001, respectively). Conversely, the second study [60] showed that a change in children’s emotional symptoms and difficulties was predicted by their worse sleep quality (β = 0.340; t = 6.87; *p* < 0.001), their increasing boredom (β = 0.23; t = 4.53; *p* < 0.001), and the mothers’ emotional symptoms and difficulties (β = 0.340; t = 4.67; *p* < 0.001).

#### 3.6.4. Associations of Emotional Symptoms with Variables Related to Family Relationships

Consistent with the significant role played by parental components in predicting a child’s emotional dysregulation, Arace and colleagues [58] found significant associations between a child’s emotional dysregulation and parental styles during the quarantine period. Parental hyperreactive and chaotic styles were positive predictors (β = 0.202, t = 5.93, *p* < 0.001 for hyperreactive and β = 0.102, t = 3.17, *p* < 0.010 for chaotic), whereas a more relaxed and balanced style was a negative predictor (β = −0.079, t = 0.33, *p* < 0.010). In addition, in the post-lockdown period, both styles, hyperreactive and chaotic, were positive predictors of a child’s anxiety and fear (β = 0.085, t = 2.45, *p* < 0.010 and β = 0.094, t = 2.87, *p* < 0.010, respectively).

The important role played by parental relationships in children‘s internalizing and externalizing behaviors during, and immediately after the COVID-19 lockdown restrictions, was investigated by Lionetti and colleagues [62] in two longitudinal studies. Study 1 carried out in preschoolers reported a significant three-way interaction effect time X parental stress X fearful temperament as a predictor of children’s externalizing behavior (*B* = 0.010, SE = 0.004, *p* < 0.005), showing that the quality of the parent–child relationship moderated children’s adjustment during the COVID-19 lockdown in relation to individual differences in temperament. At low levels of parental stress, children with low scores of fearful temperaments showed a decrease in externalizing behavior (Δ = −0.09); conversely, for children with high scores on the trait of temperament, the decrease was almost twice (Δ = −0.15). At high levels of parental stress, both groups showed an increase in externalizing behavior, regardless of their scores on the trait (Δ = −0.11 in both groups). No change emerged at medium levels of parenting stress in both groups from T1 to T2. Furthermore, a significant two-way interaction effect time*parental stress emerged as a predictor of internalizing behavior (*B =* 0.010, SE = 0.002, *p* < 0.050); in other words, the higher the parenting stress, the higher the increase in children’s internalizing behaviors from T1 to T2. The pattern of findings, which were compared with the ones observed one month after the lockdown period (T3), confirmed the three- and two-way effects in predicting children’s externalizing and internalizing behaviors, respectively. Study 2, carried out among schoolers at two time points (before the COVID-19 emergency, January 2020, T1, and one month after the lockdown, T2), reported a two-way interaction time X parent–child closeness in predicting externalizing behaviors (*B* = −0.37, SE = 0.13, *p* < 0.010). The lower the parent–child closeness, the higher the increase in children’s externalizing behaviors from T1 to T2 (Δ = 0.46, 0.20, and 0.07 for low, medium, and high values of parent–child closeness). Moreover, a three-way interaction effect time*parent–child closeness*environmental sensitivity was observed as a predictor of internalizing behavior (*B* = −0.37, SE = 0.17, *p* < 0.050), thus showing that the degree of change in internalizing behaviors from T1 to T2 was moderated by parent–child closeness. Specifically, at low levels of parent–child closeness, children with low and high scores on environmental sensitivity showed an increase from T1 to T2 (degree of change: Δ = 0.08 and −0.03, for low and highly sensitive children). At medium levels of parent–child closeness, no significant change was recorded in both groups (with low and high scores of sensitivity). At high levels of parent–child closeness, highly sensitive children showed a reduction in internalizing behaviors during the lockdown (Δ = −0.23), whereas, for lower sensitive children, levels of internalizing behaviors remained unchanged.

Similarly, the investigation carried out by Picca and colleagues [66] showed that, in younger children (1–5 years old), worsened relations between parents and children increased the risk of affective disorders (OR = 9.45, CI [4.72–18.9], *p* < 0.001), whereas improved parental relations and the remote mode of working in both parents (OR = 0.67, CI [0.46–0.97], *p* < 0.050; OR = 0.47, CI [0.30–0.72], *p* < 0.000) reduced the risk of affective disorders. In the older group (6–10 years old), both worsened parental and parent–child relationships increased the risk of irritability (OR = 1.98, CI [1.29–3.03], *p* < 0.010; OR = 7.86, CI [4.83–12.8], *p* < 0.001, respectively), and improved parent–child relationships (OR = 0.58, C.I. [0.44–0.78], *p* < 0.000) decreased the risk of irritability.

Provenzi et al.’s study [67] assessing the short-term consequences of COVID-19 pandemic-related prenatal maternal stress on infants’ temperament at 3 months tested a path model of the relationships between the study variables observed at three time points: two variables (COVID-19-related maternal stress and maternal social support) at the prenatal period during the COVID-19 lockdown (T0), one variable (maternal state anxiety) at the neonatal period (T1), and three variables (maternal parenting stress, mother–infant bonding, and infants’ temperament) at 3-month assessments in January 2021 (T2). The findings showed optimal fit indexes χ^2^_(4)_ = 5.75, *p* > 0.050; CFI = 0.990, TLI = 0.960, RMSEA = 0.052; SRMR = 0.042, suggesting the key role played by the maternal factor. Indeed, the direct path from prenatal stress (T0) to parenting stress (T2) became significant via maternal state anxiety (T1) and totally mediated the association, whereas the direct path from prenatal maternal social support (T0) to post-natal mother–infant bonding (T2) remained significant, although maternal state anxiety (T1) continued to play a significant effect (partial mediation). In addition, the relationship between maternal state anxiety (T1) and infants’ regulatory capacity (T2) was totally mediated by parenting stress and mother–infant bonding (both assessed at T2).

Finally, the study conducted by Scrimin and colleagues [69] focused on how pandemic-related variables and parental subjective experience of COVID-19-related stress could affect schoolers’ self-reported physical and emotional health and found a three-way interaction effect between SES (high vs. low), family support, and parental stress related to COVID-19. In low SES families, family support became significant when parents obtained low (−1 SD; B = −1.47, SE = 0.68, t = −2.15, *p* < 0.050) and average levels of stress in relation to COVID-19 (B = −0.98, SE = 0.38, t = −2.56, *p* < 0.010); conversely, family support was not significant when parents showed high levels of stress in relation to COVID-19 (+1 SD, B = −0.50, SE = 0.32, t = −1.56, *p* > 0.050) and was not significant across all levels of perceived COVID-19-related stress in high SES families (for low B = 0.48, SE = 0.50, t = 0.97, *p* > 0.050; for average B = −0.31, SE = 0.47, t = −0.67, *p* > 0.050, and for high B = −1.10, SE = 0.88, t = −1.25, *p* > 0.050).

#### 3.6.5. Association between Emotional States and Personality-Related Constructs

Matiz and colleagues [64] analyzed the affective repercussions emerging from the pandemic crisis on children’s personality-related factors in order to explore individual differences in developmental trajectories. In doing so, a series of comparisons were run between data (i) on children’s affective states gathered in 2014 and 2020, (ii) on children’s personality constructs collected in 2010–2011 and 2020, and (iii) on patterns of association of affective states with fear of COVID-19 experienced in 2020 by children of third, fourth, and fifth graders with low vs. high resilience personality profiles. The findings gave evidence of significant differences (i) ion the positive affect scores in females, t_(198,5)_ = 2.5, *p* < 0.050: in 2020, girls in the fourth and fifth grades reported a lower positive affect than girls with the same age in 2014 (M_2020_ = 40.4, SD = 7.5 and M_2014_ = 43.0, SD = 8.1, respectively); (ii) on the two dimensions of the personality construct, i.e., harm avoidance (HA) scores in males, t_(245,2)_ = 3.1, *p* < 0.050 and self-transcendence (ST) in the total sample, t_(494,9)_ = − 3.0, *p* < 0.050: children assessed during the pandemic showed lower HA (M_males2020_ = 7.9, SD = 4.0) and higher ST scores (M_2020_ = 5.8, SD = 2.0) than children at the same age assessed before the pandemic in 2010–2011 (M_HA-males2010–2011_ = 9.4 SD = 4.5; and M_ST-2010–2011_ = 5.2, SD = 2.1). (iii) When comparing children with high vs. low resilience (HR vs. LR) personality profiles, significant differences were found on the positive affect score (PA) (t_(158,5)_ = 2.5, *p* < 0.050), negative affect score (NA) (t_(146,9)_ = −5.4, *p* < 0.001), and fear of COVID-19 (FCV-19S) score (t_(185,2)_ = −4.9, *p* < 0.001). Children belonged to LR showed lower PA scores (M = 40.9, SD = 7.5), higher NA scores (M = 28.6, SD = 8.8), and higher FCV-19S scores (M = 13.2, SD = 3.6) in comparison with their counterparts (M_PANAS-PA_ = 43.0, SD = 6.9; M_PANAS-NA_ = 23.4, SD = 6.8; M_FCV-19S_ = 11.3, SD = 2.9).

Scaini and colleagues [68] identified temperament profiles that might constitute a potential risk factor for the development of psychopathology and low levels of resilience among school age children experiencing the COVID-19 quarantine. They reported differences between high vs. low resilient profiles (F(3) = 9.276, *p* < 0.001), high vs. low symptomatology profiles (F(3) = 19.950, *p* < 0.001), high vs. low externalizing symptomatology profiles (F(3) = 26.061, *p* < 0.001), and high vs. low internalizing symptomatology profiles (F(3) = 11.359, *p* < 0.001). With regard to resilience profiles, children who belonged to the high-resilient group showed lower scores of novelty seeking (NS; M_HighResilient_ = 6.64, SD = 3.31, *p* < 0.010; M_LowResilient_ = 8.53, SD = 3.78, *p* < 0.010) and higher scores of reward dependence (RD; M_HighResilient_ = 6.64, SD = 1.92, *p* < 0.050; M_LowResilient_ = 5.69, SD = 2.09, *p* < 0.050) and persistence (P; M_HighResilient_ = 3.31, SD = 1.77, *p* < 0.010; M_LowResilient_ = 2.21, SD = 1.53, *p* < 0.010) compared to their counterparts. Children profiled with a low symptomatology score (SDQ score) exhibited lower levels of NS (M_LowSDQ_ = 6.72, SD = 3.07, *p* < 0.010; M_HighSDQ_ = 10.43, SD = 3.84, *p* < 0.010) and higher levels of both RD (M_LowSDQ_ = 6.24, SD = 1.96, *p* < 0.050; M_HighSDQ_ = 5.34, SD = 2.17, *p* < 0.050) and P (M_LowSDQ_ = 3.05, SD = 1.61, *p* < 0.010; M_HighSDQ_ = 1.60, SD = 1.49 *p* < 0.010). Moreover, different patterns of temperamental patterns for internalizing and externalizing symptomatology were observed. Children with low levels of externalizing behavior displayed lower scores of NS (M_LowExternal_ = 6.49, SD = 2.90, *p* < 0.001; M_HighExternal_ = 11.40, SD = 3.29, *p* < 0.001) and high scores of RD (M_LowExternal_ = 6.20, SD = 1.99, *p* < 0.050; M_HighExternal_ = 5.37, SD = 2.15, *p* < 0.050) and P (M_LowExternal_ = 2.99, SD = 1.66, *p* < 0.001; M_HighExternal_ = 1.63 SD = 1.41, *p* < 0.001), whereas children with high scores of internalizing symptoms displayed higher levels of HA (M_HighInternal_ = 10.94, SD = 4.06, *p* < 0.001; M_LowInternal_ = 8.260, SD = 4.34, *p* < 0.001) and lower levels of P (M_HighInterna_ = 1.94, SD = 1.45, *p* < 0.001; M_LowInternal_ = 2.95, SD = 1.73, *p* < 0.001).

#### 3.6.6. Emotional Dysregulation and Coping Strategies

Consistent with the findings reported by Matiz and colleagues [64] related to children living in Northern Italy with a low resilience profile characterized by higher levels of both negative affect and fear of COVID-19 and lower levels of positive affect, the findings obtained by Liang and colleagues [61] showed higher levels of anxiety, mood alteration, and cognitive symptoms in children living in the same geographic areas. When facing stressful events, researchers not only examined how children consciously adjust their emotions, behaviors, and cognition by using different types of coping strategies but also tested significant differences in coping strategies between children living in Northern and Central Italy. Although the data reported no statistical differences in total scores of the three coping strategies (task-oriented, U = 138142, *p* > 0.050; emotion-oriented, U = 131008.5, *p* > 0.050; avoidance-oriented, U = 138040, *p* > 0.050), differences among some specific coping strategies were observed. For task-oriented strategies, children from Northern Italy seemed to use less humor when talking about the quarantine or COVID-19 than those from Central Italy (7.4% vs. 13.1%; χ2 = 8.759, *p* <0.010), but they were more likely to accept what was happening (66.3 % vs. 59.4%; χ2 = 5.147, *p* < 0.050). For emotion-oriented strategies, children from northern areas seemed to seek affection in others (41.3 % vs. 33.1%; χ2 = 7.627, *p* < 0.010). Finally, differences between the two groups emerged on the type (U = 124113, *p* < 0.001) and source (U = 119466, *p* < 0.001) of COVID-19 information received. Children from northern areas received more information about transmission (89.6 % vs. 82.4%; χ2 =10.785, *p* < 0.001), protection measures (90.3 % vs. 85.0 % χ2 = 6.426, *p* < 0.050), and symptoms (65.8% vs. 59.4%; χ2 = 4.467, *p* < 0.050) through TV (58.0 % vs. 51.8%; χ2 = 3.969, *p* < 0.050), whereas children of Central Italy received information from schools (33.0% vs. 59.1 %; χ2 = 70.478, *p*< 0.001).

#### 3.6.7. Emotional Dysregulation and Technology (ab)Use

Two studies [65,66] analyzed the negative effects of technologies on emotional outcomes. Indeed, Oliva et al. [65] showed that the amount of time spent (for more than two hours per day) on smartphones (estimates ranging from 1.32 to 2.41), on social media/chat and gaming (estimates equal to 2.41 and 1.82, respectively for schoolers), and watching television (estimates from 1.70 to 1.76 for pre and primary schoolers) and its increased use during the lockdown period (estimate = 2.39 for preschoolers) were risk factors for individual vulnerability to emotional and behavioral symptoms. Likewise, Picca et al. [66] recorded a higher frequency of watching videos/movies (54.4%), gaming (41%), and using chats (48.5%). The researchers also found that for older children, the increased time spent looking at smartphones/tablets and TV was significantly associated with the risk of sleep disturbances (OR = 1.32, *p* < 0.050 and OR = 1.46, *p* < 0.010, respectively) and attention disturbances (OR = 1.32, *p* < 0.050). For younger children, the use of a device for more than two hours per day significantly increased the risk of attention disorders (OR = 1.42, *p* < 0.050).

## 4. Discussion

To our knowledge, the current contribution is the first systematic review focused on the effects of lockdown on adverse emotional outcomes in Italian children (0–12 years). We emphasized the assumption that children have been impaired by a sort of dynamic network among several core aspects ranging from psychological factors in terms of dispositional, affective, cognitive, and contextual variables (personalities structures, emotion regulation, parental–child relationships, and sociodemographic variables) to environmental conditions brought on by the pandemic and the different lockdown periods. This analysis could be useful to inform health psychology science and practice.

Results generally confirmed an increased emotional dysfunction in Italian children in terms of anxiety, separation anxiety, anger, irritability, lowering of mood, difficulty in concentration, and sleep disturbances, thus underlying the complex interaction between dispositional and situational factors in outlining developmental trajectories.

The observed increased levels of negative emotions (anger and sadness) [59,60] and externalizing (difficulty falling asleep and excessive food seeking) and internalizing (difficulty standing still, concentrations problems, nervousness, and irritability) symptoms in preschoolers and schoolers [58,62,63,65] provide evidence for the cascade effect provoked by the acute social isolation experienced during the global pandemic. This is in line with prior research showing that children who had lived through quarantine presented experienced four times more distress than those who had not experienced it [72]. An investigation carried out on Chinese children during the COVID-19 pandemic reported the presence of psychological difficulties associated with feelings of fear, greater attachment to caregivers, and an increase in attention and concentration difficulties [53].

Furthermore, this systematic review highlighted the interplay of the stressful event (lockdown) with several factors, such as gender [68], age [58,66], the quality of family relationships [58,62,63,66,67,69], parental socioeconomic status [69], sleep disorders, geographical location [61,65], temperament [62,67], and individual resources (coping strategies and resilience) [62,63,64,68].

Regarding gender differences, during the lockdown, males showed more externalizing symptoms [58], which was consistent with prior studies reporting on how males were more prone to developing externalizing symptoms than females [73], although higher levels of internalizing symptoms (such as depression and general anxiety) were observed in female adolescents [74,75]. Such results on different gender effects may be related, to some extent, to different age periods, thus suggesting the assumption that specific emotions and behaviors are associated with the pubertal stage (chronological age and pubertal timing). Indeed, puberty affects girls more negatively than boys, who are over-represented in early-onset attention deficit disorder and hyperactivity. The emerging findings from this systematic review seemed to confirm that, compared to males, females are better off during childhood with respect to mental health problems, but they partly seem to lose that advantage during adolescence [76,77].

Regarding age, in line with other current international studies showing that preschoolers were likely to be more vulnerable to change in affective, anxiety, and behavioral problems rather than schoolers [78,79], it appears that for Italian preschoolers, the emotional distress rate was almost twice as high for infants and primary schoolers [60], and children aged over 3 years were more afraid of diseases since they showed higher levels of generalized and separation anxiety, somatic symptoms, and higher hunger than infants [58]. With regard to sleep disorders, one study [58] showed that higher scores of anxiety were linked to a higher prevalence of insomnia, and younger children seem to be at a higher risk of sleep disturbances during the pandemic situation, which were characterized by rapid changes in sleep processes.

The different degrees of emotional distress that emerged when looking at the three age categories (preschoolers, infants, and schoolers) could be aligned with those developmental theories and empirical results that focused on the key role played by peer relationships in the self-regulation process of internal emotional experiences [17,18,19,20]. In fact, whereas for infants, where the interactions with caregivers represent the heart of interpersonal relationships, for preschoolers, social bonding and affiliative behaviors are fundamental to regulating their own emotional process. During the pandemic situation, the effect of physical distancing, such as the deprivation of social contact with peers and the lack of touch experiences in kindergarten, might have determined social touch craving and, consequently, dysfunction in emotion regulation.

Considering this evidence, the preschool age appears to be a particularly critical period for future development trajectories and, for this reason, it represents a more reliable “neuroplastic” target than the school-age or adolescent population, in which anxiety and mood disorders have been already developed [80].

It is noteworthy that an unexpected result was found in positive emotions. Toddlers (aged 0–2 years) were happier in comparison with older children (aged 3–5 and 6–12 years), albeit younger children (aged 0–2 years) were sadder and more anxious [66]. Although the investigation was characterized by a sample size not equally distributed, since most respondents were parents of children aged between 6 and 12 years (83.3%), the unexpected result could be explained by the fact that social lockdown had initially offered infants and parents to spend more time together at home, thus increasing levels of happiness, but the prolonged time in home confinement spent by parents without working has inextricably caused their emotional upset. Through a sort of mirroring process of emotional distress in parent–infant relationships, higher levels of sadness and anxiety also emerged in children.

The supposed mirroring process of emotional distress could be confirmed when considering the role played by family relationships on children’s emotional symptoms. The Italian findings generally remarked on the importance of a good quality of the child–parent relationship in modulating the emotional impact caused by the pandemic [58,62,63,66,67,69]. For example, when looking at the association between parental well-being and children’s emotional regulation, studies gave evidence of (i) the importance of intimate bonding between younger children and caregivers in the earliest stages of development and the linkage of the risk of mood disorders with a high likelihood of worsened parent–child and parental couple relationships in both younger and older children [66]; (ii) how the maternal levels of anxiety experienced before and after birth had an impact on the regulatory capacity of three-month-old children, thus emphasizing the fact that, in families more severely affected by COVID-19, children acted as emotional barometers for their stormy relationships by picking up on emotions and mirroring them back to their parents [67]; and (iii) how parents’ individual well-being and the quality of the parental couple relationship are important in defining optimal functional family dynamics and good-quality parent–child relationships [66], which, in turn, positively affect children‘s emotional regulation and their peer interactions [81,82]. This is in line with other investigations on parent–child relationships carried out during the unprecedented circumstances of the COVID-19 pandemic [83,84]. Likewise, to further stress the meaningful role played by the familiar context, the parenting styles were also examined in relation to children’s emotional distress. Compared to more balanced parenting styles that characterized parents, who considered the lockdown as an opportunity to spend more time with babies (aged 0–2 years), the hyper-reactive and chaotic routine management styles seemed to be significantly associated with negative emotional and behavioral consequences experienced by children (aged 3–6 years) [58].

In addition, the fundamental parental role was also scrutinized in relation to the concept of resilience, thus emphasizing the risk factor played by a poor parental ability to promote resilience in offspring in predicting children’s emotional distress [63]. This was in line with a recent systematic review [85] underlining the significant role played by resilience in both caregivers and young children. Indeed, the most stressed parents find it more difficult to understand their children’s needs and respond to them appropriately [86]. As suggested by Di Giovanni and colleagues [87], high levels of parental stress fail to provide the right scaffolding to children who, not receiving appropriate clarifications or reassurances, may show higher levels of stress and emotional discomfort. In addition, significant results were observed when considering the multiple interaction effects as predictors of children’s externalizing and internalizing behaviors, thus providing further support for the interconnections among parental stress or parent–child closeness, stressful events, and the child’s temperament profile [62].

Another aspect analyzed in the present review deals with the important interconnection between parental figures and the socioeconomic condition (SES) that seemed to play a further key role in family well-being. Indeed, the study carried out by Scrimin and colleagues [68] showed that the COVID-19-related stress perceived by parents was greater in families with low SES than those with high SES. This is in line with studies that underlined how the socioeconomic condition was decisive for family well-being. Low SES increased parental distress and, consequently, the quality of relationships with children [31]. Moreover, lower SES can be associated with fewer physical and material spaces in the housing context. Regarding this point, for example, Picca et al. [66] reported that, in preschoolers, the absence of outdoor spaces, such as balconies or courtyards, significantly increased the risk of developing disorders related to the quality of sleep.

Consistent with previous findings on the bidirectional associations between sleep and emotions [88] and between the impact of sleep on emotion-related brain functions [89], another investigation [60] identified children’s sleep impairments (e.g., worse quality of sleep) and mothers‘ emotional symptoms and difficulties in emotion regulation as risk factors for future affective disorders. This is in line with a recent study that highlights how Italian parents, and in particular mothers, seem to be significantly stressed by the situation related to their children’s inadequate or disrupted sleep [90]. Therefore, in terms of health promotion, the current findings may suggest that sleep and emotion regulation function as key processes for mental health [88], and clinical psychological interventions should be considered in both aspects. Combined interventions for sleep and emotion regulation skills in childhood may be very effective in preventing psychopathologies and they may be promoted both in clinical care units and prevention contexts, such as schools.

Another interesting result that emerged from our systematic review was related to different affective responses between children in Northern and Central Italy during home confinement. Some studies [60,61] reported that compared to children living in other geographic areas, those living in Northern Italy showed a low resilience profile characterized by higher levels of negative effects, such as anxiety, mood swings, and fear of COVID-19, and lower levels of positive effects. Similar to mood alterations found in Chinese adults living in proximity to the high-risk area [91], the observed differences among Italian children could be explained by the “ripple effect” that determines varying degrees of emotional fluctuation on the basis of the different COVID-19 risk areas. In other words, the closer they are to the center of the crisis event, the higher their perception of risk and negative emotions about the event. Indeed, being that the volume of COVID-19 information received directly by TV in the north was significantly higher and much more exaggerated, children experienced an increased level of threat and panic, which in turn triggered more negative emotions (sadness, boredom, fear, irritability) and adverse behavioral changes. In addition, the study conducted by Oliva and collaborators [65] confirmed that living in Northern Italy represented a risk factor for preschoolers and schoolers.

Among the dispositional variables, a couple of studies investigated the link between emotional distress, individual resilience capacity, and personality traits [64,68], emphasizing that children with low resilience showed lower scores of positive affect and higher scores of negative affect and fear of COVID-19 [64]. Children with high resilience with less psychological symptoms of an externalizing type were characterized by high scores of persistence (P) and reward dependence (RD) and lower scores of novelty seeking (NS). Furthermore, fewer traits of persistence and higher harm avoidance (HA) characterized children with internalizing-type symptoms [68]. These results are consistent with previous data reported by Eley et al. [92] on adult subjects. The authors reported that individual differences in personality explain 39% of the variance in resilience. Specifically, the personality traits that mostly explained the observed variance were self-direction, persistence, and harm avoidance, supporting the assumption that resilience is a characteristic of the mature, responsible, optimistic, persevering, and cooperative organism. To sum up, high levels of resilience are associated with some specific skills and attitudes that constitute a protective factor for adaptation and good quality of life [93].

Moreover, the different exposure (qualitative and quantitative) to COVID-19-related information also contributed to understanding the different types of coping strategies used. Children living in northern regions used fewer task-oriented strategies and more emotion and avoidance-oriented strategies than those in central areas [55]. Indeed, in northern areas, school closure, social distancing, acute social isolation, and the overwhelming media reports brought confusion and panic and, therefore, the children used more emotion-oriented strategies to find support and protection from those close to them, as well as more avoidance strategies to cope with their parents’ anxiety and stress. The findings are consistent with other studies showing how children with emotion and avoidance-oriented coping strategies were more likely to have psychological maladjustment [94,95,96], whereas children with task-oriented strategies showed better psychological adaptability behaviors [94,97].

Lastly, some studies have identified the amount of time spent on technology (smartphone, television, and tablet) as a risk factor for the development of emotional and/or behavioral symptoms [65,66], thus highlighting the two sides of the same coin. Indeed, despite the positive use of technology in providing distance learning due to school closure, the increased time spent on digital devices has represented a risk factor for children’s vulnerability to mental health and emotional dysregulation, and it could have determined brain alterations, thus impacting sleep quality and cognitive abilities [98,99,100]. As suggested by Montag and Elhai [101], the often-neglected indirect media effects should not be overseen. Following the Affective Neuroscience Theory [102], which describes seven primary emotional systems conserved across the mammalian brain, it seemed that in times of the pandemic, the child’s experiences of those activities underlying seeking and playing circuits that were essential to engage with the world and learn to socially interact with other humans, have been restricted; conversely, those experiences underlying the systems of sadness, fear, and anger have been overstimulated due to the excess of unguarded time spent on digital screens and the dyadic mirror effect related to the emphatic reflection for the fear of COVID-19 upon parent–child interaction. Sensory play deprivation together with the lack of touch experience in social relations [21] could, therefore, cause lower emphatic skills/abilities (self-awareness, self-management, social awareness, relationship skills, and responsible decision-making) underlying the emotional regulation process, thus highlighting the fact that digital devices are useful tools if they are configured as a complementary and not as a unique option in the development of social interactions [103]. A more robust parental control of children’s use of digital devices should be decisive [100].

### Limitations

Overall, the evidence quality of the studies reviewed was deemed as being in the “Satisfactory” category. Some common methodological issues that were consistent throughout many of the analyzed studies should be mentioned. All studies used convenience sampling, thus limiting the external validity of the body of evidence reviewed, but only one study [68] has justified the sample sizes or performed the power of analysis. Therefore, the lack of this statistical test makes it difficult to interpret how much the study design was generally sensitive enough to detect the differences of interest, which limits the quality of the evidence. When examining the demographic characteristics of participants, most studies were dominated by children living in the northern regions of Italy, and in particular in Lombardy, i.e., the region characterized by the highest risk of exposure to COVID-19. Therefore, this qualitative synthesis of data provides a partial picture of the emotional symptoms experienced by Italian children. Furthermore, the adverse emotional effects on children provoked by the pandemic crisis were measured mainly by developed ad hoc questions that were referred to by parents’ perceptions. The variance in prevalence rates of mood swings could be attributed to the non-representative samples and selection bias. Additionally, the database search was conducted in March 2023, and it is possible that new data are available on this topic that have not been included in this review. Therefore, a re-run of these searches and an integration of newly published studies into the review should be conducted to broaden the emotional picture portraying children across the Italian context.

The body of evidence reviewed in this paper is mainly correlational and, therefore, causality has not been robustly established between identified risk factors and emotional vulnerability. Moreover, a longitudinal approach is needed to determine the *lasting* effects of the pandemic and track the changes in affective disorders measured before the COVID-19 pandemic (baseline) and post-pandemic period, for example three or five years later. In addition, to provide early preventive interventions in the face of potential psychopathology exacerbation in future adolescents [104], research should examine differences between high-impact areas (especially the Lombardy region) and low-impact ones that were less involved.

Despite these limitations, the current study provides the first qualitative synthesis of the impact of the COVID-19 quarantine on the emotional processes of children living in Italy. Moreover, it could be considered as a first attempt that adds empirical evidence of the impact of the exposure timing of the lockdown period combined with other co-occurring risk factors on children’s dysfunctional outcomes along with a synchronic level of their development.

## 5. Conclusions and Future Directions

To sum up, this review reports the effects of acute social isolation on dysfunctional emotional outcomes in children aged 0–12 years. Consistently with the bio-psychosocial model, this review found that emotional impairments are the product of the interaction of individual, familiar, and contextual factors. Some suggestions for health promotion interventions in preventive contexts were also provided in order to reduce long-term negative effects on childhood mental health functioning. As stated by Berk et al., “the early childhood years are a crucial time for the development of self-regulation—an array of complex mental capacities that includes impulse and emotion control, self-guidance of thought and behaviour, planning, self-reliance, and socially responsible behaviour” [105] (p. 74). For example, following a universal approach, school-based preventive interventions on functional emotion regulation, skills should be encouraged as part of the regular school-day schedule and systematically targeted to support the entire class. Such interventions could represent the so-called primary prevention actions since they include all the students without any screening procedures. In addition, inspired by secondary prevention, other activities on selected populations involving small groups of participants should be promoted to address only children with specific characteristics, such as behavioral, emotional, or academic problems.

In this regard, it will be useful to implement interventions focused on developing a better attitude during stressful situations and learning cognitive protective strategies by taking into account other psychological constructs (dispositional factors and cognitive processes) strictly related to individual response to stress. For this purpose, it is noteworthy to mention previous results on strong associations between self-conscious (i.e., internalized) emotions (shame and guilt) and undesired interpersonal outcomes including social anxiety, self-consciousness for internalizing, and feelings of anger for externalizing [106], in addition to between impairments of the executive functions of working memory and the above-mentioned emotions [107] and between socio-emotional vulnerability (assessed via personality traits such as anxiety sensitivity, intolerance to uncertainty, and tendency to ruminate), and COVID-19-associated distress (post-traumatic stress and major depressive disorders) in children and adolescents [108].

Finally, results showing different coping strategies used by children may also suggest that the government, media, parents, and schools should increase the transparency and accuracy of information on COVID-19 issues, such as the ways that the virus can be transmitted and related safety measures, to quash rumors and to reduce levels of children’s confusion and panic linked to the magnification effect of the dissemination of false information. Last but not least, findings may also stress the need to implement parental psycho-educational training to monitor the amount of time spent by children on digital devices.

## Figures and Tables

**Table 1:** Information regarding sample characteristics and results.

	N (M/F)	Age (Years) Mean Age (SD)	Region: North/Center/South	Data Collection Period	Survey (Version Parents/Child)	Results
Arace et al. [58]	945 (467/475)	0–6; 3.25 (1.38)	945/0/0	COVID-19-free period, July-Sept 2020	Ad hoc version P	Significant differences were found in problematic behaviors and emotional distress: - Main effects of the within factor (time): during the lockdown period, children reported higher levels of externalizing and internalizing behaviors, and on each increased psychosomatic symptom; - Main effects of the between factor (gender): Males were more nervous, more aggressive and hyperactive, and showed more sleep problems and anxiety to adjust to new situations; - Interaction effect (time*gender): differences in the mean score were higher in the post-lockdown period: males were more nervous (M_males_ = 2.05, SD = 0.82 vs. M_females_ = 1.85, SD = 0.78, *p* < 0.001), hyperactive (M_males_ = 2.91, SD = 0.91 vs. M_females_ = 2.87, SD = 0.87, *p* < 0.001), and showed more difficulties in separation from parental figures (M_males_ = 2.12, SD = 0.95 vs. M_females_ = 2.03, SD = 0.89, *p* < 0.050); - Age effects: children aged between 1 and 6 years were more nervous and hyperactive. Children over 3 years were more afraid of diseases, showed higher levels of generalized anxiety and somatic symptoms, and frequently manifested excessive hunger; children between 1 and 3 years showed more difficulties of separation from parental figures. Regressive behaviors and difficulties in emotion regulation during the lockdown period: - Age differences: older children (between 3 and 6 years) showed more problems in regressive autonomies, F_(2,804)_ = 8.573; *p* < 0.001, showed a greater request in eating and in going to the bathroom, and emotion regulation F_(2,897)_ = 4.154; *p* < 0.010) for the proximity of the adult at the time of falling asleep. Anxiety and fear during the post-lockdown period: - Gender differences: Higher levels of refusal to leave home in males, F_(1,914)_ = 5.603; *p* < 0.050); - Age differences: children between 3 and 6 years showed higher levels of fear or hesitation toward habitual activities, F_(2,916)_ = 3.300, *p* < 0.050). Family atmosphere and parenting style during the lockdown period: - Parents with older children (aged 3–6 years) showed a hyperreactive, F_(2,940)_ = 6.04; *p* < 0.001) and chaotic style, F(_2,940)_ = 6.48; *p* < 0.010), whereas parents with younger children showed a more balanced style, F_(2,940)_ = 9.09; *p* < 0.001) and described the lockdown as an opportunity to spend more time with babies; - Parents in working conditions during the lockdown period showed higher levels of nervousness and a hyperreactive style F_(1,943)_ = 15.67; *p* < 0.001; parents showed more difficulties with a daily routine and a chaotic style F_(1,943)_ = 3.99; *p* < 0.001. On the contrary, parents not in working conditions showed a more relaxed and balanced style, F(_1,943)_ = 21.05; *p* < 0.001. Significant associations were found between parental styles and child behaviors during the quarantine and post-lockdown period: - Children’s problematic behaviors were positively associated with a hyperreactive and chaotic parental style during the lockdown (β = 0.333, t = 10.79, *p* < 0.001 and β = 0.155, t = 5.30, *p* < 0.001, respectively) and the post-lockdown period (β = 0.308, t = 9.46, *p* < 0.001 and β = 0.119, t = 3.87, *p* < 0.001, respectively) and were negatively associated with a more relaxed and balanced style during the lockdown (β = −0.161, t = −5.27, *p* < 0.001) and in the post-lockdown period (β = −0.047, t = −1.45, *p* < 0.010); - Children’s regressive behaviors and emotion dysregulation were positively associated with hyperreactive and chaotic parental styles in both periods (during the lockdown, β =.202, t = 5.93, *p* < 0.001 and β = 0.102, t = 3.17, *p* < 0.010, respectively) and were negatively associated with a more relaxed and balanced style (during the lockdown β = −0.079, t = −0.33, *p* < 0.010); - Children’s anxiety and fear in the post-lockdown period were positively associated with hyperreactive β = 0.085, t = 2.45, *p* < 0.010 and chaotic parental styles β = 0.094, t = 2.87, *p* < 0.010).
Bacaro et al. [59]	2361 (1148/1213)	0–12, 8.1 (3.02)	1769/152/133	First lockdown	Ad hoc version P	Associations of emotional dysregulation with sleep disturbances: - The mood “Sad” was associated with less insomnia (OR = 0.93, CI [0.88–0.98], *p* < 0.050); - The moods “Anxious” and “Angry” were linked to a higher prevalence of insomnia (OR = 1.06, 95% CI [1.00–1.11], *p* < 0.050 and OR = 1.11, CI [1.07–1.17], *p* < 0.001).
Cellini et al. [60]	299 (160/139)	6–10, 7.96 (5.19)	231/11/54	First lockdown	Validated version P	- Negative effects of quarantine on children’s emotional processes: a trend of worsening in EMO, F_(1,291)_ = 3.84, *p* = 0.051, an increase in COND, F_(1,291)_ = 28.17, *p* < 0.001, in HYPER, F_(1,291)_ = 16.31, *p* < 0.001, and in the total score of the PDS, F_(1,291)_ = 24.74, *p* < 0.001; - Children’s emotional alterations and psychological difficulties were predicted by changes in children’s sleep quality (β = 0.340; t = 6.87; *p* < 0.001), perceived boredom (β = 0.23; t = 4.53; *p* < 0.001), and mothers’ psychological difficulties (β = 0.340; t = 4.67; *p* < 0.001); - Mother’s difficulties in emotion regulation covariated positively with the increase in EMO, F_(1,289)_ = 14.29, *p* < 0.001; coefficient = 0.02, SE = 0.005, t = 3.78), COND, F_(1,289)_ = 14.68, *p* < 0.001; coefficient = 0.02, SE = 0.005, t = 3.83), HYPER, F_(1,289)_ = 8.10, *p* < 0.010; coefficient = 0.01, SE = 0.004, t = 2.85), and in general with PDS, F_(1,289)_ = 22.10, *p* < 0.001; coefficient = 0.05, SE = 0.01, t = 4.70).
Liang et al. [61]	1074 (558/516)	6–12, 8.99 (5.42)	433/641/0	First lockdown	Adapted version P	Significant differences were found: - Children from northern areas were most affected than those from central areas (χ^2^ = 9.245, *p* < 0.010); - Children from Northern Italy were more worried (χ^2^ = 23.120, *p* < 0.001), more preoccupied with death (χ^2^ = 10.684, *p* < 0.001), more easily alarmed (χ^2^ = 9.074, *p* < 0.010), more afraid of COVID-19 infection (χ^2^ = 17.347, *p* < 0.001), more concerned when someone left the house (χ^2^ = 34.865, *p* < 0.001), and more bored (χ^2^ = 24.817, *p* < 0.001), and they seemed to be sadder (χ^2^ = 8.621, *p* < 0.010); - Children from Central Italy were more likely to be quiet (χ^2^ = 5.275, *p* < 0.050) and less angry (χ^2^ = 4.159, *p* < 0.050); - In terms of cognitive-oriented strategies, children from northern areas seemed to use less humor when talking about the quarantine or COVID-19 (χ^2^ = 8.759, *p* < 0.010) and were more likely to accept what was happening (χ^2^ = 5.147, *p* < 0.050); - As for emotion-oriented strategies, they seemed to seek affection from others (χ^2^ = 7.627, *p* < 0.010).
Lionetti et al. [62] *Study 1*	72 (33/39)	2–6 3.82 (1.38)		T0 and T1 (before the lockdown), T2 (during the first lockdown, April 2020), T3 (after the lockdown, June 2020)	Validated versions P	In preschoolers: - Externalizing behavior was predicted by time* parenting stress*fearful temperament (T0) (B(SE) =0.013 (0.004), *p* < 0.050): At low levels of parental stress, children with low scores on fearful temperament showed a significant decrease in externalizing behaviors (Δ = −0.09), whereas in children with high scores on this trait, the decrease was almost twice (Δ = −0.15). At medium levels of parental stress, both groups showed no change. At high levels of parental stress, both groups with high and low temperaments showed an increased level (Δ = 0.11). - Internalizing behavior was predicted by the time* parenting stress interaction (B(SE) = 0.01 (0.002), *p* < 0.050). The degree of change from T1 to T2 was Δ = −0.03 for low values of parenting stress, Δ = 0.02 for medium values of parenting stress, and Δ = 0.08 for high levels of parenting stress. The results were overall stable between T1 and T3 (one month after the lockdown). A significant three-way interaction effect emerged, with time (T1-T2) X parenting stress X fearful temperament in predicting externalizing behaviors B(SE) = 0.014 (0.006), *p* < 0.050. A significant two-way interaction effect of time (T1-T2) X parenting in predicting internalizing behaviors B(SE) = 0.010 (0.003), *p* < 0.005.
Lionetti et al. [62] *Study 2*	94 (45/49)	8–10 9.08 (0.56)		T1 (before the lockdown, January 2020), T2 (during the lockdown, April 2020)	Validated versions P	In schoolers: - Externalizing behavior was predicted by a two-way interaction effect: time*parent–child closeness interaction (B(SE) =−0.37 (0.13), *p* < 0.010). From T1 to T2, significant changes were observed: for low values of parent–child closeness (Δ = 0.46) and for medium values (Δ = 0.20), whereas for high values, no changes were significant (Δ = −0.07, *p* > 0.050). - Internalizing behavior was predicted by a three-way interaction effect: time*parent–child closeness*environmental sensitivity. At low levels of parent–child closeness, significant changes were observed from T1 to T2 in children with both low and high sensitivity levels (Δ = 0.15 and 0.18). At medium values of parent–child closeness, for both low and highly sensitive children, internalizing behaviors overall did not change for any groups (Δ = 0.08 for low and = −0.03 for highly sensitive children; not significant). At high levels of parent–child closeness, highly sensitive children showed a reduction in internalizing behaviors (Δ = −0.23), while no change was reported in children with lower sensitivity levels (Δ = 0.02).
Mariani Wigley et al. [63]	158 (72/82)	6–11 8.88 (1.41)	-	The first lockdown after the one-month restriction, May–June 2020	Ad hoc version P	Significant differences were found: -Compared with past stress-related behavior, child stress-related behavior experienced during the lockdown was significantly increased in terms of difficulty standing still t_(157, 2)_ = −6.21, *p* < 0.001 (M_BeforeLockdown_ = 1.53, SD = 0.63; M_DuringLockdown_ = 1.82, SD = 0.73) concentration difficulties t_(157,2)_ = −8.07, *p* < 0.001 (M_BeforeLockdown_ = 1.65, SD = 0.58; M_DuringLockdown_ = 2.07, SD = 0.69), nervousness and irritability t_(157,2)_ = −7.63, *p* < 0.001 (M_BeforeLockdown_ = 1.61, SD = 0.54; M_DuringLockdown_ = 1.99, SD = 0.68), tendency to cry for no reason t_(157, 2)_ = −5.60, *p* < 0.001 (M_BeforeLockdown_ = 1.26, SD = 0.45; M_During Lockdown_ = 1.54, SD = 0.72), food refusal t_(157, 2)_ = −7.28, *p* < 0.001 (M_BeforeLockdown_ = 1.13, SD = 0.33; M_During Lockdown_ = 1.23, SD = 0.47), excessive food seeking t_(157,2)_ = −4.01, *p* < 0.001 (M_BeforeLockdown_ = 1.20, SD = 0.45; M_DuringLockdown_ = 1.39, SD = 0.66), difficulty falling asleep t_(157,2)_ = −3.68, *p* < 0.001 (M_BeforeLockdown_ = 1.27, SD = 0.52; M_DuringLockdown_ = 1.72, SD = 0.77), and restless sleep with awakening t_(157,2_) = −4.19, *p* < 0.001 (M_BeforeLockdown_ = 1.22, SD = 0.47; M_DuringLockdown_ = 1.38, SD = 0.58). Parents’ ability to support and promote child-resilient behaviors predicted child stress-related behaviors during the lockdown (B(SE) = −0.178 (0.069), *p* < 0.050 and mediated the associations between parents’ resilience and children’s stress-related responses to the lockdown (B(SE) = −0.002, *p* <0.001) and bootstrap 95% C.I.:−0.0048 −0.0003). The direct effect was not significant.
Matiz et al. [64]	323 (180/143)	8–12, 9.13 (0.87)	323/0/0	Second wave of infection, 2020 (Oct-Nov 2020)	Validated version C	Significant differences were found: - On positive affect scores in females, t_(198,5)_ = 2.5, *p* < 0.050: in 2020, girls reported a lower positive affect than girls in 2014 (M_2020_ = 40.4, SD = 7.5 and M_2014_ = 43.0, SD = 8.1); - On harm avoidance (HA) scores in males, t_(245,2)_ = 3.1, *p* < 0.050, M_males2020_ = 7.9, SD = 4.0, and on self-transcendence (ST) in the total sample, t_(494,9)_ = − 3.0, *p* < 0.050 (M_2020_ = 5.8, SD = 2.0); - Compared to children with high resilience profiles, children with low resilience profiles showed lower scores on positive affect (M_LR_PANAS-PA_ = 40.9, SD = 7.5; M_HR_PANAS-PA_ = 43.0, SD = 6.9), higher scores on negative affect (M_LR_PANAS-NA_ = 28.6, SD = 8.8; M_HR_PANAS-NA_ = 23.4, SD = 6.8), and higher scores on fear of COVID-19 (M_LR_FCV-19S_ = 13.2, SD = 3.6; M _HR_FCV-19S_ = 11.3, SD = 2.9).
Oliva et al. [65]	9,688 5,066/4,622	0–18 < 1 (*n* = 860) Preschool (*n* = 6,402) Primary/middle school (*n* = 2.205) >14 (*n* = 221)	4,892/2,205/2,591	The first lockdown (one month after restriction, May 2020)	Validated version P Validated version C	Emotional and behavioral status: -An increase in symptoms (35% for infants < 1 year; 73.3% for preschool children; 40.5% for primary school children; 60% for middle school children) during the lockdown; -In infants, the presence of a sibling was a protective factor for inflexibility, estimate = −1.27, SE = 0.22, *p* < 0.001, for irritability, estimate= −0.77, SE = 0.17, *p* < 0.001, and for routine, estimate = −0.56, SE = 0.17, *p* < 0.010; -In preschoolers, among demographics, the presence of sibling was a protective factor (estimate = −0.50, SE = 0.18, *p* <0.010). Being male (estimate = 0.88, SE = 0.17, *p* <0.001), living in Northern Italy (estimate = 0.66, SE = 0.18, *p* < 0.001), and a high parental educational level (estimate = 0.51, SE = 0.19, *p* <0.010) were risk factors; among the lifestyle changes, the use of a smartphone >2 h/day (estimate = 1.32, SE = 0.29, *p* < 0.001) and its increased use (estimate = 2.33, SE = 0.18, *p* < 0.001), watching television >2 h/day (estimate = 1.70, SE = 0.16, *p* <0.001) and its increased use during the lockdown (estimate = 2.39, SE = 0.16, *p* <0.001) were risk factors; - In schoolers, among demographics, being male (estimate = 1.50, SE = 0.55, *p* <0.010) and living in Northern Italy (estimate = 1.92, SE = 0.56, *p* <0.001) were risk factors; among lifestyle changes, watching videos or TV series >2 h/day (estimate = 3.06 SE = 0.81 *p* <0.001), using social media/chat >2 h/day (estimate = 2.41, SE = 0.70, *p* <0.001), gaming with electronic devices alone >2 h/day (estimate = 1.82, SE = 0.81 *p* < 0.020), and watching TV >2 h/day (estimate = 1.76, SE = 0.75 *p* < 0.020) were risk factors; physical activity (estimate = −5.80, SE = 0.92, *p* <0.001), homeschooling (estimate = −2.80, SE = 0.56, *p* < 0.001), reading a book (estimate = −2.47, SE = 0.84, *p* <0.010), and talking with other people (estimate = −1.83, SE = 0.56, *p* < 0.001) were considered protective factors. Depressive symptoms in schoolers: - Among demographics, living in northern areas (estimate = 1.20, SE = 0.40, *p* < 0.010) and parental job loss (estimate = 1.68, SE = 0.57, *p*< 0.010) were positively associated with depressive symptoms; among lifestyle changes, the risk factors were time spent on social media/chat (estimate = 1.66, SE = 0.53, *p* <0.010) and watching TV series/movies on digital devices (estimate = 1.89. SE = 0.48. *p* < 0.001). Physical activity (estimate = −3.01, SE = 0.64, *p* <0.001), talking with other people (estimate = −1.36, SE = 0.40, *p* <0.001), and playing with parents (estimate = −1.05, SE = 0.40, *p* <0.010) were protective activities. Anxiety symptoms in schoolers: - Among demographic characteristics, the presence of siblings (estimate = −1.41, SE = 0.65, *p* <0.010) was confirmed to be a protective factor. - Among lifestyle changes, physical activity (estimate = −4.38, SE =1.07, *p* < 0.001), reading (estimate = −2.00, SE = 0.95, *p* < 0.010), and talking with other people in person (estimate = −1.77, SE = 0.79, *p* < 0.010) were protective factors. Time spent on social media/chat (estimate = 2.72. SE = 0.48. *p* < 0.001) and watching TV series/movies on digital devices (estimate = 2.33. SE = 0.94. *p* < 0.001) were risk factors.
Picca et al. [66]	3392 (1764/1628)	Young vs. old children	3392/0/0	COVID-19-free period, July-Aug 2020	Ad hoc version P	- In younger children (1–5 years old; YC), a high risk of mood disorders was associated with worsened relations between parents and children (OR = 9.45, CI [4.72–18.9], *p* < 0.001), a low risk of affective disorders was related to improved parental relations (OR = 0.67, CI [0.46–0.97, *p* < 0.050) and a remote mode of working in both parents (OR = 0.47, CI [0.30–0.72], *p* < 0.000); - In older children (6–10 years old; OC) the risk of irritability was related to both worsened parental and parent–child relationships (OR = 1.98, C.I. [1.29–3.03], *p* < 0.010; OR = 7.86, CI [4.83–12.8], *p* < 0.001), whereas improved parent–child relationships (OR = 0.58, C.I. [0.44–0.78], *p* < 0.000) decreased the risk of irritability. - In OC, an increase in the time spent on screen and watching TV enhanced the risk of sleep disturbances (OR = 1.32, *p* <0.050; OR = 1.46, *p* < 0.010, respectively). An increase in the time spent watching TV was associated with the risk of attention disturbances (OR = 1.32, *p* <0.050). - In YC, the use of a device for more than 2 h per day significantly increased the risk of attention disorders (OR = 1.42, *p* <0.050).
Provenzi et al. [67]	163 (80/83)	3 months	-	First and second waves of infection, 2020 T0 (prenatal period-before the lockdown), T1 (neonatal period during the lockdown), T2 (infants’ age 3 months until the lockdown, second wave, January 2021)	Validated version P	Significant associations were found: - Infant regulatory capacity score was positively related to prenatal maternal social support (r = 0.227, *p* <0.001) and postnatal mother–infant bonding (*r* = 0.312, *p* < 0.001), and was negatively related to postnatal maternal state anxiety (*r* = −0.189, *p* < 0.050) and postnatal maternal parenting stress (*r* = −0.328 *p* <0.001); - Maternal anxiety played a mediating role in the relationship between prenatal stress and parenting stress at 3 months (total mediation) and in the relationship between prenatal support and mother–infant bonding (partial mediation); - Parenting stress and mother–infant bonding totally mediated the relationship between maternal anxiety and infants’ regulatory capacity at 3 months.
Scaini et al. [68]	158 (76/82)	5–10 7.18 (1.79)	135/15/8	COVID-19-free period (June 2020)	Validated version P	Significant differences emerged: - Between high-resilient and low-resilient profiles (F(3) = 9.276, *p* < 0.001). High-resilient children showed lower novelty seeking scores (NS; M_HighResilient_ = 6.64, SD = 3.31, *p* < 0.010; M_LowResilient_ = 8.53, SD = 3.78, *p* < 0.010) and higher reward dependence (RD; M_HighResilient_ = 6.64, SD = 1.92, *p* < 0.050; M_LowResilient_ = 5.69, SD = 2.09, *p* < 0.050) and Persistence scores (P; M_HighResilient_ = 3.31, SD = 1.77, *p* < 0.010; M_LowResilient_ = 2.21, SD = 1.53, *p* < 0.010); - Between high and low symptomatology profiles (F(3) = 19.950, *p* < 0.001). Children with low symptomatology scores exhibited lower levels of NS (M_LowSDQ_ = 6.72, SD = 3.07, *p* < 0.010; M_HighSDQ_ = 10.43, SD = 3.84, *p* < 0.010) and higher levels of both RD (M_LowSDQ_ = 6.24, SD = 1.96, *p* < 0.050; M_HighSDQ_ = 5.34, SD = 2.17, *p* < 0.050) and P (M_LowSDQ_ = 3.05, SD = 1.61, *p* < 0.010; M_HighSDQ_ = 1.60, SD = 1.49, *p* < 0.010); - Between high and low externalizing symptomatology profiles (F(3) = 26.061, *p* < 0.001). Children with low scores on the externalizing sub-scale showed the same temperament profile as those with low scored on the SDQ total score, with lower NS (M_LowExternal_ = 6.49, SD = 2.90, *p* < 0.001; M_HighExternal_ = 11.40, SD = 3.29, *p* < 0.001) and higher RD (M_LowExternal_ = 6.20, SD = 1.99, *p* < 0.050; M_HighExternal_ = 5.37, SD = 2.15, *p* < 0.050) and P (M_LowExternal_ = 2.99, SD = 1.66, *p* < 0.001; M_HighExternal_ = 1.63 SD = 1.41, *p* < 0.001). - Between high and low internalizing symptomatology profiles (F(3)= 11.36, *p* < 0.001). Vhildren with high scored on internalizing symptoms displayed higher levels of HA (M_HighInternal_ = 10.94, SD = 4.06, *p* < 0.001; M_LowInternal_ = 8.260, SD = 4.34, *p* < 0.001) and lower levels on P (M_HighInterna_ = 1.94, SD = 1.45, *p* < 0.001; M_LowInternal_ = 2.95, SD = 1.73, *p* < 0.001).
Scrimin et al. [69]	116 (61/55)	6–11, 8.70 (1.33)	-	The first lockdown, one month after restriction (May 2020)	Validated version C	Significant differences were found: - Compared with high SES families, low SES families showed greater stress-related COVID-19 uncertainty, t_(114,2)_ = 3.98, *p* < 0.005 (M_lowSES_ = 3.51, SD = 0.89; M_highSES_ = 2.88, SD = 0.82) greater stress-related COVID-19 fear, t_(114,2_) = 3.29, *p* < 0.005 (M_lowSES_ = 2.78, SD = 1.0; M_highSES_ = 2.19, SD = 0.89), and less stress-related online schooling, t_(114,2)_ = 1.23, *p* < 0.050 (M_lowSES_ = 1.63, SD = 0.94; M_highSES_ =2.83, SD = 0.80). In high SES families, children’s discomfort was associated with COVID-19 danger (*r*= 0.27, *p* < 0.050), stress related to dealing with new routines (r= 0.33, *p* < 0.050), stress related to social relationships (*r*= 0.31, *p* < 0.050), stress related to online schooling (*r* = 0.38, *p* < 0.010), stress related to COVID-19 uncertainty (*r* = 0.41, *p* < 0.010), and stress related with COVID-19 fear (*r* = 0.37, *p* < 0.010). In low SES families, children, discomfort was associated with stress related to social relationships (*r* = 0.28, *p* < 0.050) and stress related to COVID-19 fear (*r* = 0.29, *p* < 0.050). Children’s physical and emotional discomfort was predicted directly by SES (B (SE) = −17.91 (7.25), t = −2.25, *p* < 0.050), stress lockdown (B (SE) = −0.005 (0.023), t = −1.557, *p* < 0.010), and family support (B(SE) = −4.81 (1.78), t = −2.69, *p* < 0.050). Children’s physical and emotional discomfort was predicted by SES X family support X stress-related COVID-19 interaction (B (SE) = −1.53 (0.75), t = −2.03, *p* < 0.050). In low SES families, family support was significant when parents perceived low (−1 SD; B = −1.47, SE = 0.68, t = −2.15, *p* < 0.050) and average stress in relation to COVID-19 (B = −0.98, SE = 0.38, t = −2.56, *p* < 0.010), which was not significant when the levels of COVID-19 stress were high. In high SES families, family support was not significant for COVID-19-related stress.

## Data Availability

No new data were created or analyzed in this study. Data sharing is not applicable to this article.

## Supplementary Materials

The following supporting information can be downloaded at https://www.mdpi.com/article/10.3390/ijerph20126168/s1, Table S1: Quality of appraisal of the included studies assessed with the Newcastle–Ottawa Scale–Cross-sectional studies [36,58,59,60,61,63,64,65,66,68,69] (n = 10). Table S2: Quality of appraisal of the included studies assessed with the Newcastle–Ottawa Scale–Longitudinal studies [35,62,67] (n = 3).
