# Supplementary material for: Emotions in Times of Pandemic Crisis among Italian Children: A Systematic Review
Source: Int J Environ Res Public Health. 2023 Jun 18;20(12):6168. doi: 10.3390/ijerph20126168 (PMC10297924; doi:10.3390/ijerph20126168)
Supplement: Supplementary file 1 [file ijerph-20-06168-s001.zip › ijerph-2387534-supplementary.pdf]

International Journal of
Environmental Research and Public Health

MDPI

Table S1. Quality of appraisal of the included studies assessed with the Newcastle–Ottawa Scale–Cross-sectional studies [36] (n=10).

	**Selection Domain**				**Comparability Domain**	**Outcome Domain**		**Score (0-10)***
	Sample representativeness	Sample size	Non-response rate	Exposure definition	Adjustment of the outcome	Assessment of the outcome	Statistical test	
Arace et al. [58]	0	1	1	1	1	1	1	6
Bacaro et al. [59]	1	1	0	1	1	1	1	6
Cellini et al. [60]	1	1	1	2	1	1	1	8
Liang et al. [61]	1	1	1	2	1	1	1	8
Mariani Wigley et al. [63]	0	1	1	2	1	1	1	7
Matiz et al. [64]	0	1	1	2	0	1	1	6
Oliva et al.[65]	1	1	1	2	1	1	1	8
Picca et al. [66]	1	1	1	1	1	1	1	7
Scaini et al.[68]	1	1	1	2	1	1	1	8
Scrimin et al.[69]	0	1	1	2	1	1	1	7
Average Domain	4.2				0.9	2		7.1

* 9–10 =Very Good; 7–8 =Good; 5–6 =Satisfactory; 0– 4 = Unsatisfactory. This scale has been adapted from the Newcastle–Ottawa Quality Assessment Scale for cohort studies and the adapted Newcastle–Ottawa scale created for cross-sectional studies.

Table S2. Quality of appraisal of the included studies assessed with the Newcastle–Ottawa Scale–Longitudinal studies [35] (n=3).

	**Selection Domain**				**Comparability Domain**	**Outcome Domain**			**Score (0-9)***
	Representativeness of exposed cohort	Selection of non-exposed cohort	Ascertainment of exposure	Outcome of interest at start of study	Adjustment of the outcome	Assessment of the outcome	Follow-up length	Adequacy of follow up	
Lionetti et al. *Study 1* [62]	1	0	0	1	1	0	1	1	5
Lionetti et al. *Study 2* [62]	1	0	0	1	1	0	1	1	5
Provenzi et al [67]	1	0	0	1	1	0	1	1	5
Average Domain	2				1	2			5

* 9–10 =Very Good; 7–8 =Good; 5–6 =Satisfactory; 0– 4 = Unsatisfactory. This scale has been adapted from the Newcastle–Ottawa Quality Assessment Scale for cohort studies and the adapted Newcastle–Ottawa scale created for cross-sectional studies.
